# Detection of *Escherichia coli* and Associated β-Lactamases Genes from Diabetic Foot Ulcers by Multiplex PCR and Molecular Modeling and Docking of SHV-1, TEM-1, and OXA-1 β-Lactamases with Clindamycin and Piperacillin-Tazobactam

**DOI:** 10.1371/journal.pone.0068234

**Published:** 2013-07-04

**Authors:** Shailesh K. Shahi, Vinay K. Singh, Ashok Kumar

**Affiliations:** School of Biotechnology, Faculty of Science, Banaras Hindu University, Varanasi, Uttar Pradesh, India; Aligarh Muslim University, India

## Abstract

Diabetic foot ulcer (DFU) is a common and devastating complication in diabetes. Antimicrobial resistance mediated by extended-spectrum β-lactamases (ESBLs) production by bacteria is considered to be a major threat for foot amputation. The present study deals with the detection of *Escherichia coli* and the prevalence of *bla*_TEM_, *bla*_SHV_ and *bla*_OXA_ genes directly from biopsy and swab of foot ulcers of diabetic patients. In total, 116 DFU patients were screened, of which 42 suffering with severe DFUs were selected for this study. Altogether 16 *E. coli* strains were successfully isolated from biopsy and/or swab samples of 15 (35.71%) patients. ESBL production was noted in 12 (75%) strains. Amplification of β-lactamase genes by multiplex PCR showed the presence of *bla*_CTX-M_ like genes in 10 strains, *bla*_TEM_ and *bla*_OXA_ in 9 strains each, and *bla*_SHV_ in 8 of the total 16 strains of *E. coli.* Out of the ten antibiotics tested, *E. coli* strains were found to be resistant to ampicillin (75%), cefoxitin (56.25%), cefazolin (50%), meropenem (37.5%), cefoperazone (25%), cefepime (31.25%), ceftazidime (56.25%), and cefotaxime (68.75%) but all showed sensitivity (100%) to clindamycin and piperacillin-tazobactam. 3D models of the most prevalent variants of β-lactamases namely TEM-1, SHV-1, OXA-1, and ESBL namely CTX-M-15 were predicted and docking was performed with clindamycin and piperacillin-tazobactam to reveal the molecular basis of drug sensitivity. Docking showed the best docking score with significant interactions, forming hydrogen bond, Van der Waals and polar level interaction with active site residues. Findings of the present study may provide useful insights for the development of new antibiotic drugs and may also prevent ESBLs-mediated resistance problem in DFU. The novel multiplex PCR assay designed in this study may be routinely used in clinical diagnostics of *E. coli* and associated *bla*_TEM_, *bla*_SHV,_ and *bla*_OXA_ like genes.

## Introduction

Diabetic foot infections (DFIs) are common, often resulting in potentially devastating complications in diabetic patients. DFIs are associated with high morbidity and risk of lower extremity amputation [1]. Limb amputation has a major impact on the individual, not only in distorting the body structure, but also with regard to loss of productivity, increasing dependency, and costs of treating foot ulcers if patients require inpatient care [2]. Wound infection, delay in wound healing, neuropathy and ischemia in combination with a foot ulcer are the most common causes of diabetes-related amputations [3]. Patients with diabetes are more likely to lose a limb than those without diabetes and up to eighty-five percent of lower-limb amputations in patients with diabetes are preceded by infected foot ulceration [3]. To meaningfully describe and evaluate the severity of foot ulcer, several systems of classification are currently in use [4], [5]. Wagner’s system of classification is routinely used in determining the surgical intervention to foot ulcer on admission. In more superficial infections which are classified according to Wagner (Wagner grades I-II), aerobic gram-positive bacteria are the predominant organisms. In deeper wounds (Wagner grades III-V), gram-negative bacteria are frequently found [6]. *Escherichia coli*, *Proteus* spp., *Pseudomonas* spp., *Staphylococcus aureus* and *Enterococcus* spp. are the most frequent pathogens contributing to progressive and widespread tissue destruction [7]. In the patients with DFI, there is a predominance of *E. coli* (24.20%) and antibiotic resistance is wide spread in this species [8]. The predominant mechanism of resistance to β-lactams in *E. coli* is production of extended-spectrum β-lactamases (ESBLs). Bacterial strains producing these enzymes inactivate the drugs by hydrolyzing the β-lactam ring [9]. ESBLs -producing bacteria are emerging as a worldwide clinical threat. In the early 1960s, *bla*_TEM-1_ was the first plasmid-mediated β-lactamase gene in *E. coli* [10]. Subsequently, another common β-lactamase gene *bla*_SHV-1_ was reported from *Klebsiella pneumoniae* and *E. coli.* Various new β-lactam antibiotics have been developed since 1960s for the treatment of patients which have resulted in emergence of other ESBL. Different types of β-lactamase have been reported during the 1990s however, TEM- and SHV-types are more common [11]. During the past decade, rapid and massive spread of CTX-M-type ESBLs have been reported. These enzymes are now the most prevalent ESBLs in Enterobacteriaceae and also occur rarely in *Pseudomonas* spp. and *Acinetobacter baumannii* [12] in Europe and in other parts of the world [13]. The list of ESBLs is increasing and the total number of well characterized ESBLs exceeds 200 [11].

The main objective of this study was to develop a simple and rapid method for the detection of *E. coli* isolates and associated β-lactamase genes (*bla*_TEM_, *bla*_SHV_, and *bla*_OXA_) from patients suffering from DFI. Attempt was made to predict three dimensional (3D) model of TEM-1, SHV-1, OXA-1 (β-lactamases) and CTX-M-15 (ESBL). Furthermore, the identification of the amino acid residues crucial to the interaction between selected β-lactamases with clindamycin and piperacillin tazobactum was performed. Additionally, docking studies of TEM-1, SHV-1, OXA-1 and CTX-M-15 proteins with clindamycin and piperacillin-tazobactum were performed. It is anticipated that modeling and docking studies may be useful in developing new class of drugs to control ESBLs-mediated antibiotic resistance problem in DFUs.

## Materials and Methods

### Patients and Sample Collection

This study was conducted in the School of Biotechnology. Samples and details of patients were obtained from the Department of Endocrinology and Metabolism, and the Department of General Surgery, Sir Sunderlal Hospital, Institute of Medical Sciences, Banaras Hindu University, Varanasi. Approval of the institutional ethics committee of Banaras Hindu University (Ref. No. Dean/2009-10/555 dated July 11, 2009) was obtained to conduct this study. Prior written consent was also obtained from every recruited patient. In total, 116 diabetic foot patients attending to the hospital between January 2010 and October 2011 were screened and 42 suffering with severe DFIs (Wagener’s grade III-V) were selected for the study. Grading of DFUs was done according to Wagner [4].

Tissue samples from infected DFUs were obtained from the ulcer using a sterilized 6 mm punch biopsy needle under local anaesthesia. Two swab and tissue samples from each patient were collected by washing the wound with sterile physiological saline. One swab and tissue sample was used for detecting *E. coli* through *in vitro* culture, the second set of sample was used for detecting *E. coli* by PCR.

### Isolation and Identification of *E. coli*

A direct smear was made from each sample (swab and biopsy) and plated directly onto MacConkey agar. The inoculated plates were immediately placed in an aerobic environment and incubated at 35°C for 24 h. The plates were examined after 24h of incubation and distinct pink colonies that appeared on each plate were picked up and restreaked on respective media. Tentative identification of *E. coli* was made on the basis of Gram’s staining, morphological characteristics, and biochemical tests namely, catalase, urease, Simmons citrate utilization and MR (methyl red) as per the standard methods. *E. coli* JM109 (Promega, USA) was used as reference strain.

### Isolation of Genomic DNA

Genomic DNA of swab and biopsy samples was extracted using a fast tissue PCR Kit (MBI Fermentas, USA). Genomic DNA from the laboratory-grown cultures was isolated using a DNeasy tissue kit (Qiagen, Germany) according to the instructions of the manufacturer. Plasmid DNA from *E. coli* strains was isolated using a PureLink HiPure plasmid miniprep kit (Invitrogen, USA) according to the instructions of the manufacturer.

### Primer Designing, Amplification and Sequencing of *E. coli* Specific 16S rDNA

Primer3 (http://frodo.wi.mit.edu/) tool was used for designing *E. coli* gene specific primers from species-specific region (*16S rRNA* dimethyladenosine transferase). 16S rDNA (1467 bp) was amplified from the template DNA of the reference strain *E. coli* JM109, strains of *E. coli* isolated from DFUs, and biopsy/swab samples of DFUs. Amplification was performed in a final volume of 50 µl containing 1×PCR assay buffer with 1.5 mM MgCl_2,_ 25 pmol of each primers (Fd.5′-TGTGGGAACGGCGAGTCGGAATAC-3′ and Rev 5′GGGCGCAGGGGATGAAACTCAAC-3′) (Integrated DNA Technologies, USA), 250 µmol each of the dNTPs, 1U *Taq* DNA polymerase (Bangalore Genei, Banhalore) and 100 ng of template DNA. Conditions for PCR amplification were; initial denaturation for 10 min at 94°C, 30 cycles of 40 s at 94°C, 40 s at 60°C and 1 min at 72°C followed by final extension of 7 min at 72°C. 5 µl of the amplified PCR product was electrophoresed on a 2% agarose gel in Tris-borate-EDTA buffer (TBE) containing ethidium bromide (0.5 µg/ml) and monitored in gel documentation unit (BioRad Laboratories, USA). 16S rDNA (1467 bp) amplified from *E. coli* (isolated from DFU) was sequenced to confirm the identity and to confirm the specificity of primers. Additionally, the specificity of primer was confirmed using template DNA from other gram-negative bacteria *viz*., *Klebsiella* spp., *Enterobacter* spp. *Citrobacter* spp, *Serratia* spp., and *Pseudomonas* spp. Based on sequence similarity, the representative isolate was identified as *E. coli* strain DF39TA. The sequence was submitted to NCBI database under accession number JX017293.

### Antibiotic Susceptibility Testing

Antimicrobial susceptibility was done by the disc diffusion method using the Kirby-Bauer method [14]. Ten antibiotics belonging to four broad classes namely, cephalosporins: cefazolin (30 µg), cefoxitin (30 µg), cefoperazone (75 µg), cefepime (30 µg) ceftazidime (30 µg), and cefotaxime (30 µg); penicillins: piperacillin/tazobactam (100/10 µg) and ampicillin (10 µg); lincosamides: clindamycin (2 µg), and carbapenems: meropenem (10 µg) were tested. These antibiotics were selected according to previously published recommendations and their widespread use in treatment of various diseases [7]. Interpretation of results was according to the CLSI guidelines 2010 [15].

### Phenotypic Detection of ESBL and Carbapenemases

ESBL phenotype of various isolates was determined by double disc diffusion synergy test (DDST). Briefly, equal amount of inoculum from each isolate was added to Mueller Hinton broth and grown for 24 h at 37°C. 100 µl of broth culture (approx. 10^6^ cells/ml) was uniformly spread onto sterile Mueller Hinton Agar. Antibiotic discs of amoxicillin/clavulanic acid (20/10 µg) and cefotaxime (30 µg), and ceftazidime (30 µg), were placed at a distance of 15 mm apart and plates were incubated at 37°C overnight. Enhancement of zone of inhibition of any of the cephalosporins towards the amoxycillin/clavulanic acid disc was considered as ESBL producer [16]. The *E. coli* isolates which were found to be positive for ESBL phenotype were subjected to E-test.

E-test for confirming the ESBL phenotype was performed according to Coudron et al. [17]. ESBL results were considered positive if the isolates had an MIC (µg/ml) of ≥1 for ceftazidime (CAZ), ≥0.5 for cefotaxime (CTX), and the ratio for CAZ-CLA and CTX-CTL was more than or equal to 8 [17], [18]. *E. coli* ATCC strain 25922 and *Klebsiella pneumoniae* ATCC strain 700603 (HiMedia, Mumbai, India) were used as negative and positive controls for ESBL production test respectively. *E. coli* strains that showed a zone diameter of 16–21 mm for meropenem were tested for carbapenemase production by Modified Hodge test (MHT) as per the CLSI recommendation [19].

### Multiplex PCR and Sequencing of *bla*_TEM_, *bla*_SHV_, *bla*_OXA,_ and *16S rRNA* Genes of *E. coli*

Multiplex PCR was performed in a single tube with primers of *bla*_TEM_, *bla*_SHV_, *bla*_OXA_ and *16S rRNA* genes. PCR assay was performed in a total volume of 50 µl which contained; 25 pmol of the primers of *16S rRNA* (Fd 5′-TGTGGGAACGGCGAGTCGGAATAC-3′ and Rev 5′-GGGCGCAGGGGATGAAACTCAAC-3′), 10 pmol primers of each of the *bla*_TEM_, *bla*_SHV_, and *bla*_OXA_ as described by Dallenne et al. [20], 200 µM each of the dNTPs, 1 U of *Taq* DNA polymerase, 1×PCR assay buffer with 1.5 mM MgCl_2_ and 100 ng of template DNA or 5 µl of macerated biopsy samples. PCR conditions were used as described by Dallenne et al. [20]. PCR was run in a PTC-100 Thermal Cycler (MJ Research, Inc., USA). 5 µl of the amplified PCR product was used for electrophoresis and visualization was made as mentioned above. Multiplex PCR was also performed separately for *bla*_CTX-M Gp1_, *bla*_CTX-M Gp2_ and *bla*_CTX-M Gp9_ genes as described previously [20].

Amplified product of *bla*_TEM_, *bla*_SHV_, *bla*_OXA,_ and *bla*_CTX-M_ genes was purified by QIAquick gel extraction kit (Qiagen, Hilden, Germany) according to the instructions of the manufacturer. Sequencing and homology search of the amplified products were done on commercial basis from Chromous Biotech PVT Ltd., Bangalore, India. After complete annotation, the sequences were submitted to NCBI database (http://www.ncbi.nlm.nih.gov/) and all accession numbers are shown in Table S1.

### Gene Annotation and Similarity Search

Sequences of *bla*_TEM-1_, *bla*_SHV-1_, *bla*_OXA-1,_ and *bla*_CTX-M*-*15_ genes from *E. coli* strain DF39TA were subjected to ORF scan (http://www.ncbi.nlm.nih.gov/gorf/gorf.html) to identify coding regions (exons). FGENESB was used to predict operons and genes in raw sequences [21]. The predicted putative protein sequences were subjected to protein functional analysis using INTERPROSCAN version 4.4 [22]. These protein sequences were used for homology search. Similar sequences from different species were retrieved and aligned using ClustalW [23]. Phylogenetic tree was constructed using the UPGMA method and tree was inferred by bootstrap phylogenetic inference using MEGA4 [24]. The conserved motifs present in these sequences were analyzed using BLOCKS and MEME (multiple EM for motif elicitation) software version 3.5.7 [25]. For motif analysis, the selection of maximum number of motifs was set to 10 with minimum width of 10 amino acids, while for genes a maximum number of motifs were set to 20 while other factors were default selections for putative proteins.

### Retrieval of the Target Protein Sequence and Template Identification

Predicted putative protein sequences of *bla*_TEM-1_, *bla*_SHV-1_, *bla*_OXA-1,_ and *bla*_CTX-M*-*15_ genes were used as target for homology modeling. Discovery studio 3.1 [26], [27] was used for comparative homology modeling of TEM-1, SHV-1, OXA-1, and CTX-M-15 protein using template structures. PDB advance BLAST (http://www.rcsb.org/pdb/home/home.do) was applied for template identification to construct 3D models of the target proteins.

### Model Refinement and Evaluation

Predicted 3D models of TEM-1, SHV-1, OXA-1, and CTX-M-15 proteins were used for evaluation and refinement. 3D model was subjected to energy minimization using the steepest descent technique to eliminate bad contacts between protein atoms. Computations were also carried out in vacuo with the GROMOS96 43B1 parameters with Swiss Pdb Viewer (http://expasy.org/spdbv/) tool. The backbone conformation of the predicted model was inspected using the Phi/Psi Ramachandran plot of PDBSum database (http://www.ebi. ac.uk/pdbsum/) [28] and RAMPAGE server (http://mordred.bioc.cam.ac.uk/~rapper/rampage.php) [29]. The SUPERPOSE server [30] (http://wishart.biology.ualberta.ca/SuperPose/) was employed to perform structural alignment between the target and template structure. ProSA and ERRAT servers (https://prosa.services.came.sbg.ac.at/prosa.phphttp://nihserver.mbi.ucla.edu/ERATv2/) were used for model quality evaluation. Evaluated and refined models were deposited to Protein Model DataBase (PMDB; http://mi.caspur.it/PMDB/).

### Active Site Prediction and Docking

After obtaining the final model, the possible binding sites of TEM-1, SHV-1, OXA-1, and CTX-M-15 proteins were searched using Q-SiteFinder (http://bmbpcu36.leeds.ac.uk/qsitefinder/). Ten binding sites were obtained for TEM-1, SHV-1, OXA-1, and CTX-M-15. These binding sites were compared to the active site of the template to determine the residues forming the binding pocket. Clindamycin (C_18_H_33_C_l_N_2_O_5_S{(2S,4R)-N-[2-chloro-1-[(2R,3R,4S,5R,6R)-3,4,5-trihyd roxy 6-methyl sulfan yloxan-2-yl] propyl]-1-methyl-4 propylpyrrolidine-2-carboxamide), and piperacillin-tazobactam (C_33_H_39_N_9_O_12_S_2_ {(2R,5R,6R)-6-[[2-[(4-ethyl-2,3-dioxopiperazine-1-carbon yl)amino]-2phenylacetyl]amino]-3,3-dimethyl-7-oxo-4-thia-1-azabicyclo[3.2.0]heptane-2-carboxylic acid;(2S,3S,5R)-3-methyl-4,4,7-trioxo-3-(triazol-1-ylmethyl)-4λ6-thia-1-azabicyclo[3.2.0]heptane-2-carboxylic acid} were used for docking study. These inhibitors were docked using LibDock module of Discovery studio 3.1. Docking calculations and interactions were analyzed using DS visualizer.

## Results and Discussion

### Isolation and Identification of *E. coli*

Sixteen strains of *E. coli* were successfully isolated from biopsy/swab samples of 15 out of 42 patients (35.71%) admitted to the S.S Hospital, Varanasi. Two *E. coli* strains were isolated from DFU of one patient (DF30). Identity of *E. coli* strains was confirmed by morphological characteristics, biochemical tests and amplification of the *E. coli* specific 16S rDNA. Similar to our findings 32.07% of the DFU patients from south India were reported to carry infection of *E. coli* in DFUs [31]. Alavi et al. [32] also reported that *E. coli* (23.8%) was the most predominant gram-negative organisms in DFUs from patients of Iran.

One of the interesting outcomes of the study was use of species specific primers for the identification of bacteria from DFUs. All the strains of *E. coli* isolated from DFUs showed amplification of the desired fragment of 16S rDNA (1476 bp) with *E. coli* specific primers in PCR assay. Interestingly, DNA isolated from 42 biopsy/swab samples also resulted in amplification of *E. coli* specific amplicon in 8 biopsy and 7 swab samples. However, template DNA isolated from other gram-negative bacteria did not show amplification of *E. coli* specific amplicon. As the PCR-based results matched with the laboratory-grown cultures, it is concluded that direct diagnosis of *E. coli* and/or other species of bacteria by PCR is possible directly from biopsy/swab samples. That these strains indeed belonged to *E. coli* also became evident from the sequencing of amplified product from a representative strain of *E. coli*. The sequences showed 99% similarity with sequences available in the NCBI for *E. coli*.

### Antibiotic Resistance Profiles

Antibiotic sensitivity test revealed that all the sixteen *E. coli* strains of DFUs show high percentage of resistance to a number of antibiotics. Prevalence of resistance among the isolates was; ampicillin (75%), cefoxitin (56.25%), cefazolin (50%), meropenem (37.5%), cefoperazone (25%), cefepime (31.25%) ceftazidime (56.25%), and cefotaxime (68.75%). However, all the strains showed sensitivity (100%) to clindamycin and piperacillin/tazobactam. Similar to our findings, occurrence of multiple antibiotics resistance has been reported in several bacteria but only few reports are available for bacteria isolated from DFUs [31]–[33]. Sensitivity of *E. coli* to clindamycin and piperacillin/tazobactam has also been reported by other researchers previously [34], [35]. Sana et al. [36] reported that 82.2% isolates of *E. coli* were susceptible to piperacillin/tazobactam. Similarly, a study conducted at Mahatma Gandhi Medical College and Research Institute, Pondicherry, reported that the members of Enterobacteriaceae were mostly susceptible to tazobactam [35].

### ESBL and Carbapenemase Production in *E. coli*

Of the 16 *E. coli* isolates, 12 (75%) were ESBL producers according to the results of phenotypic tests DDST and E test (Table 1). ESBL-producing strains were found to be resistant to β -lactam antibiotics namely, ampicillin (83%), cefazolin (50%), cefoperazone (25%), cefepime (33.33%), cefoxitin (58.33%), ceftazidime (75%), and cefotaxime (91.66%). Additionally, six isolates (37.5%) showed resistance to meropenem which seems uncommon for *E. coli* species. Surprisingly, all these six isolates did not show the presence of carbapenemase by MHT. Recently, Shanmugam et al. [37] and Sahu et al. [38] have reported as high as 45.4% and 37% strains of *E. coli* resistant to meropenem respectively. With the available data, it is indeed hard to assign the exact mechanism of resistance to meropenem, it would be essential to confirm the presence of various types of carbapenemase genes employing PCR assay. This is the shortcoming of the present study and needs further investigation.

**Table 1:** Details of resistance phenotypic and genetic characteristics of the *E. coli* strains.

Serial No.	*E. coli* strains	Cefotaxime	Ceftazidime	ESBL DDST	Etest for confirmation		ESBL			
					CAZ/CLA	CTX/CTL	TEM	SHV	OXA	CTX-M
1	DF3SB	S	S	−	−	−	−	−	−	−
2	DF5SC	R	S	+	−	+	TEM-1	SHV-12	OXA-1	CTX-M-9
3	DF6SA	R	R	+	+	+	TEM-1	−	OXA-1	CTX-M-15
4	DF7SA	R	R	+	+	+	−	SHV-12	OXA-1	CTX-M-15
5	DF9SB	R	R	+	+	+	TEM-1	−	OXA-1	CTX-M-15
6	DF39TA	R	R	+	+	+	TEM-1	SHV-1	OXA-1	CTX-M-15
7	DF13TB	R	S	+	−	+	−	SHV-5	−	CTX-M-1
8	DF18SA	S	S	−	−	−	−	SHV-1	OXA-1	−
9	DF29TA	R	R	+	+	+	TEM-20	−	OXA-1	CTX-M-15
10	DF10TB	R	S	+	−	+	−	−	OXA-1	−
11	DF30TA	R	R	+	+	+	TEM-20	SHV-1	−	CTX-M-15
12	DF30TD	R	R	+	+	+	TEM-52	SHV-1	−	CTX-M-15
13	DF31TA	S	S	−	−	−	−	−	−	−
14	DF40TA	S	R	+	+	+	TEM-10	SHV-2	−	−
15	DF43TD	S	S	−	−	−	−	−	−	−
16	DF49SA	R	R	+	+	+	TEM-1	−	OXA-1	CTX-M-3

Available reports dealing with the prevalence of ESBL producers amongst various isolates of *E. coli* show marked variations [7], [33]–[36]. In fact, the prevalence of ESBL producing *E. coli* isolates show significant differences among geographical locations within India [34], and other parts of world ranging from 0% in Iceland to less than 1% in Estonia, 41% in Romania, 16.8% in Iran [35], 25.2% in Tiruchirapali, South India [39], and 31.86% in Turkey [40]. Gadepalli et al. [7] reported 54.5% *E. coli* isolates as ESBL producers in a tertiary care hospital in New Delhi. Detection of ESBL producing strains of *E. coli* is of vital importance as they are responsible for the spread of resistance among different bacteria. A combination of factors such as co-selection due to MDR phenotypes, virulence factors, mobile genetic elements, clonal spread of virulent strains and the acquisition of diverse ESBL-bearing plasmids may facilitate the spread of ESBL and other resistances [41].

### Occurrence of *bla*_TEM_, *bla*_SHV_, *bla*_OXA,_ and *bla*_CTX-M_ Genes and Comparative Analysis of the ESBL Phenotype

**Figure 1:**
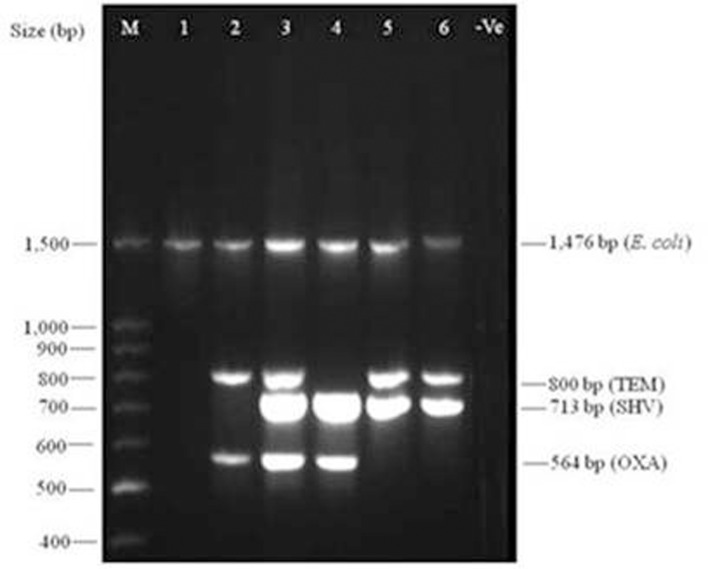
Representative multiplex PCR for the amplification of *bla*_TEM_, *bla*_SHV_, *bla*_OXA_, and 16S *rRNA* genes from culture of *E. coli* and biopsy and swab samples of 5 patients. Lanes 1–6; 1- *E. coli* JM109, 2-DF6S, 3-DF5S, 4-DF7S, 5-DF30T, and 6-DF40T, –Ve negative control (without template). M-100bp marker (New England Biolabs, USA). Template from the biopsy/swab samples also showed amplification depending upon the presence of respective genes.

Multiplex PCR assay was employed to detect the prevalence of *bla*_TEM_, *bla*_SHV,_ and *bla*_OXA_ like genes as well as *E. coli* in a single PCR reaction. Typical representation of multiplex PCR for simultaneous amplification of *bla*_TEM_, *bla*_SHV_, *bla*_OXA,_ and 16S *rRNA* genes from 5 (2 biopsy and 3 swab) samples are shown in Figure 1. The amplified products were identical to those obtained by pure culture of *E. coli*. That the amplified products are indeed originating from strains of *E. coli* became evident from the fact that all the strains showed amplification of *E. coli* specific amplicon similar to the reference strain, *E. coli* (JM109) (Figure 1). *bla*_SHV_ like gene was detected in 8 of the 16 *E. coli* positive DFUs. Further analysis of *bla*_SHV_ gene revealed that four strains of *E. coli* possess *bla*_SHV-1_, two *bla*_SHV-12_, one each has *bla*_SHV-5_ and *bla*_SHV-2_ (Table 1). *bla*_TEM_ was detected in 9 *E. coli* strains of which five isolates expressed *bla*_TEM-1_, two *bla*_TEM-20_ and one each showed expression of *bla*_TEM-52_ and *bla*_TEM*-*10_ (Table 1). Interestingly, *bla*_OXA_ like gene was noted in 9 strains of *E. coli* of which all produced *bla*_OXA-1_ (Table 1).

Multiplex PCR was also performed separately to detect the prevalence of *bla*_CTX-M_ like gene. Of the 16 pure cultures of *E. coli* strains, presence of *bla*_CTX-M_ like gene was noted in 10 strains (62.5%) (Table 1). Further analysis revealed that *bla*_CTX-M-15_ was the most widespread among different strains of *E. coli* (7/16 strains, 43.47%), followed by *bla*_CTX-M-9_, *bla*_CTX-M-3,_ and *bla*_CTXM*-*1_ which were present in each one strain (1/16 strains, 6.25%). However, the predominance of CTX-M-15-producing *E. coli* in this study may be due to the virulent ST131 clone and the diverse plasmids bearing the *bla*_CTX-M-15_ gene. Further studies are needed to decipher the genetic traits responsible for showing CTX-M-15 predominance in this study. Sequences of *bla*_TEM_ (TEM-1, -10, -20, and -52), *bla*_SHV_ (SHV-1, -2, -5, and 12), *bla*_OXA_ (OXA-1), and *bla*_CTX-M_ (CTX-M-1, -3, -9, and -15) - type genes were submitted to NCBI database and accession numbers are shown in Table S1. Occurrence of several β-lactamases genes has been reported, but *bla*_TEM_, *bla*_SHV_, *bla*_OXA,_ and *bla*_CTX-M_ -type ESBLs genes are the most predominant [10]. Kiratisin et al. [42] screened 235 strains of ESBLs producing *E. coli* and reported that 87.3%, 77% and 3.8% of the strains were carriers of the *bla*_CTX-M_, *bla*_TEM,_ and *bla*_SHV_ genes respectively. A few strains were found to carry the *bla*_OXA_ gene. The OXA β-lactamases, known as oxacillinases are equally important as they degrade isoxazolyl β-lactams such as oxacillin and methicillin. OXA enzymes belong to the Class D group of β-lactamases and are known to be present in a number of bacteria [43]. SHV, TEM, and CTX-M enzymes belong to Ambler class A and were initially reported as plasmid borne in gram-positive bacteria [44]. More than 200 types of well characterized β-lactamases enzymes have been reported [45] and several attempts have been made to categorize them since the late 1960s [46]–[49].

Comparative analysis of the phenotypic results of three ESBL tests revealed some discrepancies due to different substrate profiles of the different ESBLs. The overall phenotypic results obtained with different ESBL tests and the multiplex PCR amplification assay were in good agreement (Table 1). Of the 16 *E. coli* isolates, 12 (75%) were ESBL producer as per the results of phenotypic test comprising DDST and E-test (Table 1). Of 12 ESBL producing strains, 10 showed ESBL phenotype due to the presence of either *bla*_CTX-M_ (CTX-M-1, -3, -9, and -15), *bla*_TEM_ (TEM-10, -20, and -52) and *bla*_SHV_ (SHV-2, -5, and -12) genes. One strain (DF40TA) showed ESBL phenotype due to the presence of *bla*_TEM-10_ and *bla*_SHV-2_ ESBL genes (Table 1). *E. coli* strain DF10TB showed ESBL phenotype but ESBL gene was absent. ESBL positive phenotype of DF10TB strain may be due to the presence of other ESBL-types (CTX-M-8 like, CTX-M-25 like, PER, GES, VEB) or by spontaneous mutation in *amp*D gene that codes for an amidase and induces constitutive overproduction of the AmpC enzyme leading to increased resistance to ESBLs such as oxyiminocephalosporins (cefotaxime, ceftriaxone and ceftazidime).

### Gene Annotation, Similarity Search and Phylogenetic Analysis

Genes, *bla*_OXA-1_ (Class D, Genbank Id: JX294482.1, Genpept Id: AFR79064.1), *bla*_SHV-1_ (Class A, Genbank Id: JX294483.1, Genpept Id: AFR79065.1) *bla*_TEM-1_ (Class A, Genbank Id: JX294484.1, Genpept Id: AFR79066.1) and *bla*_CTX-M-15_ (Class A, Genbank Id: JX294480.1, Genpept Id: AFR79062.1) were used for alignment and phylogenetic classification with similar bacterial proteins. INTERPROSCAN study revealed that the SHV-1, TEM-1, and CTX-M-15 showed similarity with three domains namely, IPR000871, IPR012338 and IPR023650 of β-lactamase family. However, OXA-1 β-lactamases showed similarity with two domains viz. IPR001460 (penicillin-binding protein) and IPR012338 (β-lactamase domain). Using these identified proteins, BLAST (basic local alignment search tool) search was performed to find homologous regions in the sequences of different bacterial species available in the biological database (NCBI). Multiple sequence alignment revealed highly conserved regions in *bla*_OXA-1_, *bla*_SHV-1_, *bla*_TEM-1,_ and *bla*_CTX-M-15_ genes (Figures S1a-S4a). Phylogenetic analysis among different species against these four genes showed closest neighbors with at least 98% similarity (Figures S1b-S4b). *E. coli bla*_OXA-1_ gene alignment indicated multilevel consensus sequence against different organisms, *viz*. *Klebsiella oxytoca*, *Shigella flexneri*, *Klebsiella pneumoniae*, *Proteus* sp., *Pseudomonas aeruginosa*, *Salmonella enterica* (Figure S1b) whereas *bla*_SHV-1_ gene alignment revealed multilevel consensus sequence with *Klebsiella pneumoniae*, *Klebsiella oxytoca*, *Shigella dysenteriae*, *Enterococcus faecalis*, *Enterobacter asburiae* (Figure S2b). Similarly, *bla*_TEM-1_ gene showed highest percentage of similarity with *Klebsiella pneumoniae*, *Serratia marcescens*, *Acinetobacter baumannii*, *Morganella morganii*, *Enterobacter cloacae*, *Proteus mirabilis*, and *Pseudomonas aeruginosa* species (Figure S3b). The alignment of *bla*_CTX-M-15_ gene showed multilevel consensus sequence against *Shigella boydii, Enterobacter gergoviae, Morganella morganii, Klebsiella pneumoniae, Citrobacter freundii, Shigella sonnei* and *Proteus mirabilis* (Figure S4b). However, one multilevel consensus motif (RPDERFPMMSTFKVWLCGAV) was present in the protein sequences of all the genes (Figure S5).

### Homology Modeling of OXA-1, SHV-1, TEM-1, and CTX-M-15 Proteins

**Figure 2:**
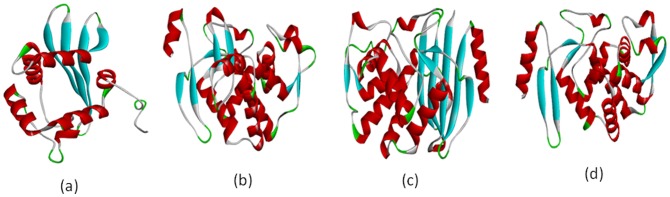
Three dimensional models of OXA-1 (a), SHV-1 (b), TEM-1 (c), and CTX-M-15 (d).

The homology modeling of OXA-1, SHV-1, TEM-1, and CTX-M-15 proteins was performed with Discovery studio 3.1 and is represented in Figure 2. Additionally, the 3D model of these proteins was constructed using the PDB BLAST for template identification. Analyses revealed that OXA-1 shares 99% similarity as well as positivity with PDB code: 1M6K of *E. coli* [50]. Similarly, SHV-1 showed 99% similarity and 100% positivity with the PDB code: 3D4F crystal structure of *Klebsiella pneumoniae* [51]. TEM-1 showed 94% similarity and 94% positivity with PDB code: 1ERM of *E. coli* [52]. In the case of *bla*_CTXM-15_, there were 86% similarity and 93% positivity with PDB code: 2ZQ8 crystal structure of *E. coli*. Electrostatic energy of predicted OXA-1, SHV-1, TEM-1, and CTX-M-15 models were -5659.92, -7887.66, -9139.24, and -7430.49 kcal/mol respectively as per the analysis done by CHARMm force field of Discovery studio 3.1. Based on simulation study, it became evident that the predicted models are highly stable. Details of modeling and simulation results for OXA-1, SHV-1, TEM-1, and CTX-M-15 are available in Table S2.

### Model Assessment (Refinement and Evaluation)

Stereochemical quality of the predicted protein structure was assessed using RAMPAGE and PDBSum. The Ramachandran plot study of OXA-1, SHV-1, TEM-1, and CTX-M-15 revealed that more than 90% of residues were in favoured regions having good stereochemical quality. Analysis of OXA-1, SHV-1, and CTX-M-15 revealed that 93.5, 98.1, and 98.9% residues occur in the favoured region respectively, with no residues present in the outlier region. TEM-1 showed 98.3% residues in favoured region and 0.4% residues in outlier region (Table S3). No residues were observed in the disallowed region. Further analysis based on PDBSum showed that the residues present in the most favoured regions for OXA-1, SHV-1, TEM-1, and CTX-M-15 were 87.7, 95.2, 95.7, and 95.3% respectively (Table 2). ERRAT score for the models of OXA-1, SHV-1, TEM-1, and CTX-M-15 was 91.15, 82.12, 92.30, and 92.35 respectively which are well within normal range for a high quality model. The best refined and validated structures of OXA-1, SHV-1, TEM-1, and CTX-M-15 were deposited in the PMDB database with PMDB-IDs; PM0078526, PM0078524, PM0078525, and PM0078527 respectively. The weighted root mean square deviation (RMSD) of the Cα trace between the template and the final refined model of OXA-1, SHV-1, TEM-1, and CTX-M-15 showed that the target and the template structures are closely similar at the backbone and at the CA tom level, yielding a significant Z-score. Resolution of the predicted OXA-1, SHV-1, TEM-1, and CTX-M-15 structure showed significant resolution of 2.363, 1.677, 2.021, and 1.62Å respectively using RESPROX server. Structure quality estimation using PROSA showed significant Z-scores of -6.04, -5.27, -7.66, and -6.38 for OXA-1, SHV-1, TEM-1, and CTX-M-15 respectively as compared to the template Z-score. Structure quality estimation using QMEAN also resulted in significant Z-score (Table S4). The modeled structures of OXA-1, SHV-1, TEM-1, and CTX-M-15 revealed that each monomer belongs to the alpha-beta: 3-layer (aba) sandwich DD-peptidase/β-lactamase super family (3.40.710.10) (Figure S6). Secondary element composition is also available in Table S5.

**Table 2:** Stereo-chemical properties using PDBSum.

Proteins	% Residue in mostfavoured regions	% Residue in additional allowed regions	% Residue in generously allowed regions	% Residue in disallowed regions
OXA-1	87.7	11.0	0.7	0.0
SHV-1	95.2	4.3	0.5	0.0
TEM-1	95.7	3.8	0.5	0.0
CTX-M-15	95.3	4.1	0.6	0.0

### Active Site Prediction of OXA-1, SHV-1, TEM-1, and CTX-M-15 Proteins

**Figure 3:**
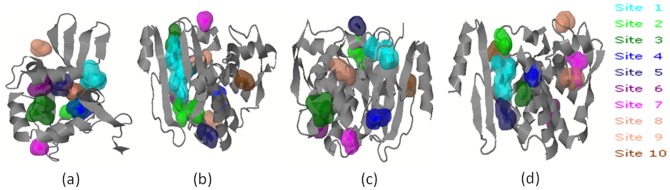
Active site prediction of OXA-1 (a), SHV-1 (b), TEM-1 (c), and CTX-M-15 (d).

Among the ten binding sites obtained from Q-Site finder (Figure 3), site 1 was found highly conserved in OXA-1, SHV-1, and CTX-M-15 and therefore selected as the active site for docking study with clindamycin and piperacillin-tazobactam. However, sites 3 and 7 were found highly conserved in TEM-1 and thus used for docking purpose. The results of multiple sequence alignment and active site prediction revealed that the residues in site 1 of OXA-1 (LYS^16^, THR^17^, MET^19^, GLN^20^, GLU^103^, ASN^104^, MET^105^, TYR^106^, LEU^107^, GLY^118^, LYS^119^, THR^120^ and PHE^135^) were identical to the reported active site of *E. coli* template structure. Similarly, residues in site 1 of SHV-1 (ARG^12^, VAL^13^, GLY^14^, LEU^15^, ILE^16^, PHE^35^, PRO^36^, MET^37^, MET ^38^, THR^40^, THR^150^, THR^151^, PRO^152^, MET^155^, ALA^156^, LEU^159^, ARG^209^, GLY^210^, ALA^211^, ARG^212^, GLY^213^, ILE^214^_,_ and VAL^215^) were similar to the active site of *K. pneumoniae* template [51]. In the case of CTX-M-15, residues in site 1 (ARG^24^, LEU^25^, GLY^26^, VAL^27^, ALA^28^, GLU^44^, PHE^46^, ALA^47^, MET^48^, THR^51^, THR^161^, SER^162^, PRO^163^, ARG^164^, MET^166^, ALA^167^, and LEU^170^ ) showed similarity with 2ZQ8 - Apo structure of class a β-lactamase from *E. coli*. Similar to our study, Shakil and Khan [53] have also reported docking between CTX-M-15 of *E. coli* with cefotaxime.

In case of TEM-1, site 3 (ALA^53^, VAL^54^, SER^56^, ARG^57^, GLN^62^, THR^115^, ILE^116^, GLU^121^, LEU^122^, ALA^124^, PHE^125^, ASN^128^ ), and site 7 (ASN^128^, MET^129^, LYS^166^, LEU^167^, GLY^170^, GLU^171^_,_ and LEU^172^) were found conserved and showed homology with the active site of TEM-1 β-lactamase from *E. coli* [52]. All the 10 possible active sites for OXA-1, SHV-1, TEM-1, and CTX-M-15 models are available in Table S6. All these putative predicted active sites show close similarity with the reported active sites of respective β-lactamases [51]–[53] and support our findings.

### Molecular Docking of OXA-1, SHV-1, TEM-1, and CTX-M-15 Proteins

Docking of OXA-1, SHV-1, TEM-1, and CTX-M-15 protein was performed with clindamycin and piperacillin-tazobactam. Final docked conformations obtained for these inhibitors were evaluated considering the number of hydrogen bonds formed and the bond distance between atomic co-ordinates of the active site and inhibitor.

**Figure 4:**
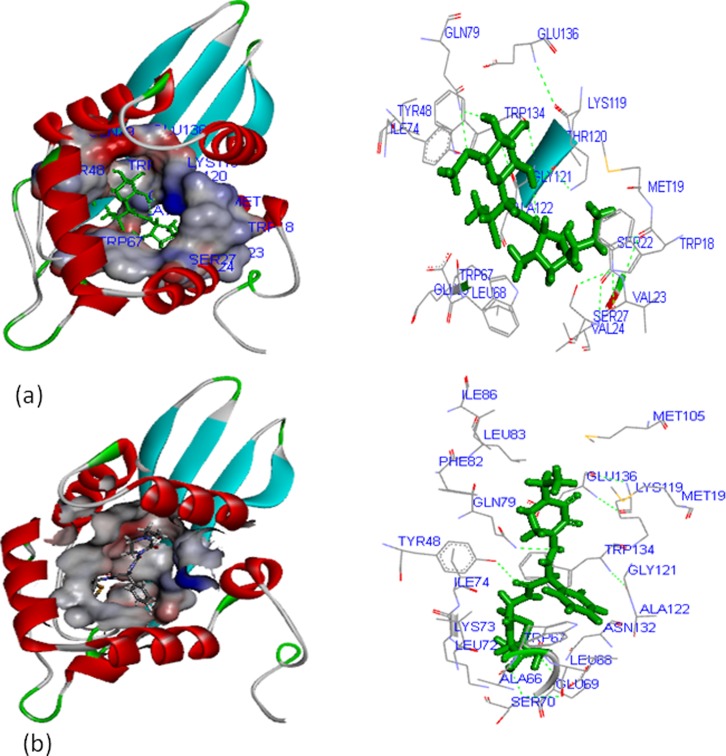
Interaction of OXA-1 with; (a) clindamycin, and (b) piperacillin-tazobactam.

With OXA-1 it was noted that clindamycin and piperacillin-tazobactam interacted with the major active site cavity, with site volume of 234 cubic Å. The residues TRP^18^, MET^19^, Ser^22^, VAL^23^, VAL^24^, SER^27^, TYR^48^, TRP^67^, LeU^68^, GLU^69^, ILE^74^,GLN^79^, LYS^119^, THR^120^, GLY^121^, ALA^122^, TRP^134^_,_ and GLU^136^ were mainly involved in the interaction with clindamycin (Figure 4a) whereas piperacillin-tazobactam interacted with MET^19^, TYR^48^, ALA^66^, TRP^67^, LEU^68^, GLU^69^, SER^70^, LEU^72^, LYS^73^, ILE^74^, GLN^79^, PHE^82^, LEU^83^, ILE^86^, MET^105^, LYS^119^, GLY^121^, ALA^122^, ASN^132^, TRP^134^, and GLU^136^ (Figure 4b). Validity of docking is corroborated from the fact that MET^19^, MET^105^, LYS^119^_,_ and THR^120^ residues are commonly reported as prominent active site of OXA-1 protein for both, clindamycin and piperacillin-tazobactam. Our findings also show that the binding orientation of both the compounds is proper with the inhibitory active domain.

**Figure 5:**
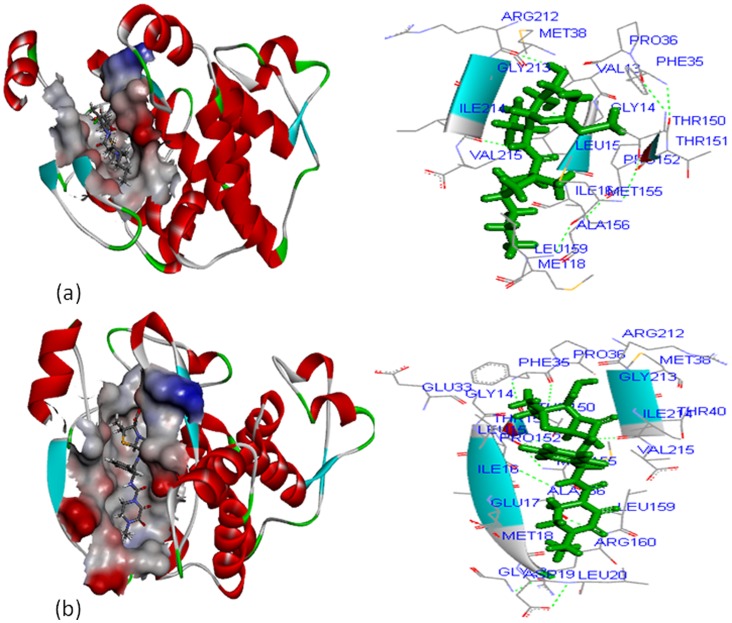
SHV-1 interaction with; (a) clindamycin, and (b) piperacillin-tazobactam.

In the case of SHV-1 protein, clindamycin and piperacillin-tazobactam interacted with the major active site cavity with a site volume of 486 cubic Å. The residues namely, VAL^13^, GLY^14^, LEU^15^, ILE^16^, MET^18^, PHE^35^, PRO^36^, MET^38^, THR^150^, THR^151^, PRO^152^, MET^155^, ALA^156^, LEU^159^, ARG^212^, GLY^213^, ILE^214^, and VAL^215^ were involved in interaction with clindamycin (Figure 5a) whereas GLY^14^, LEU^15^, ILE^16^, GLU^17^, MET^18^, ASP^19^, LEU^20^, GLY^23^, GLU^33^, PHE^35^, PRO^36^, MET^38^, THR^40^, THR^150^, THR^151^, PRO^152^, MET^155^, ALA^156^, LEU^159^, ARG^160^, ARG^212^, GLY^213^, ILE^214^_,_ and VAL^215^ interacted with piperacillin-tazobactam (Figure 5b). For both the protein complexes VAL^13^, GLY^14^, LEU^15^, ILE^16^, MET^18^, ASP^19^, LEU^20^, GLY^23^, GLU^33^, PHE^35^, PRO^36^, MET^38^, THR^40^,THR^150^, THR^151^, PRO^152^, MET^155^, ALA^156^, LEU^159^, ARG^160^, ARG^212^, GLY^213^, ILE^214^, and VAL^215^ residues seem to be the prominent active site.

**Figure 6:**
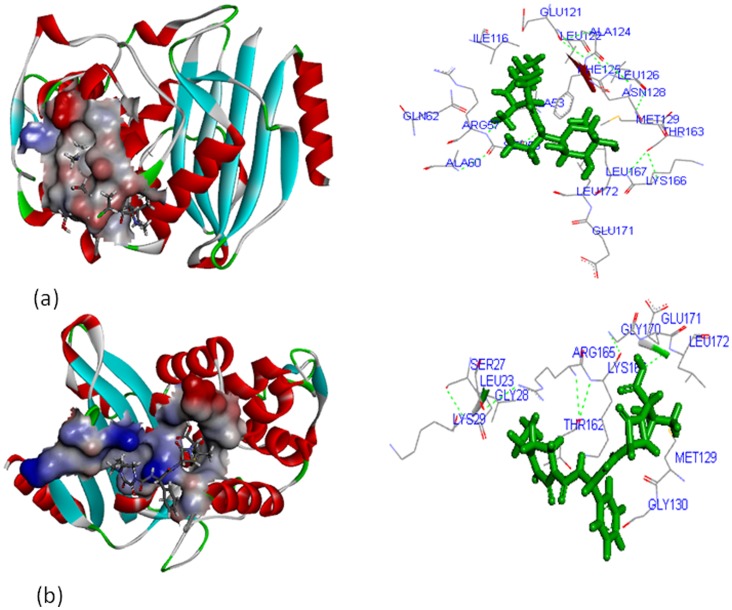
Interaction of TEM-1 with; (a) clindamycin, and (b) piperacillin-tazobactam.

For the TEM-1 protein, clindamycin and piperacillin-tazobactam showed interaction with the major active site cavity (sites 3 and 7) with site volume of 216 and 90 cubic Å respectively. The residues ALA^53^, SER^56^, ARG^57^, ALA^60^, GLN^62^, ILE^116^, GLU^121^, LEU^122^, ALA^124^, PHE^125^, LEU^126^, ASN^128^, MET^129^, THR^163^, LYS^166^, LEU^167^, and LEU^172^, were involved in interaction with clindamycin (Figure 6a) whereas LYS^16^, LEU^23^, SER^27^, GLY^28^, LYS^29^, MET^129^, GLY^130^, THR^162^, ARG^165^, GLY^170^, GLU^171^, and LEU^172^ interacted with piperacillin-tazobactam (Figure 6b). In both the complexes ALA^53^, SER^56^, ARG^57^, GLN^62^, ILE^116^, GLU^121^, LEU^122^, ALA^124^, PHE^125^, ASN^128^, THR^163^, GLY^170^, GLU^171^_,_ and LEU^172^ residues were found to be the prominent active sites.

**Figure 7:**
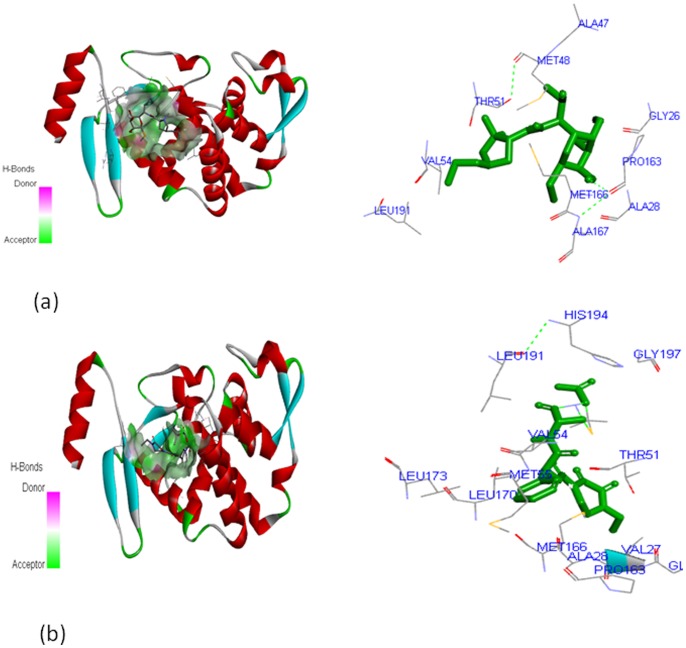
Interaction of CTX-M-15 with; (a) clindamycin, and (b) piperacillin-tazobactam.

In the case of CTX-M-15 protein, clindamycin and piperacillin-tazobactam showed interaction with the major active site cavity (with site volume of 255 cubic Å). The residues namely, GLY^26^, ALA^28^, ALA^47^, MET^48^, THR^51^, VLA^54^, PRO^63^, MET^166^, ALA^167^_,_ and LEU^191^ showed interaction with clindamycin (Figure 7a), ALA^26^, VAL^27^, THR^51^, VAL^54^, MET^55^, LEU^173^, MET^166^, PRO^163^, LEU^191^_,_ and HIS^197^ interacted with piperacillin-tazobactam (Figure 7b). For both the protein complexes GLY^26^, ALA^28^, THR^51^, PRO^163^, MET^166^, LEU^170^_,_ and HIS^194^ residues were found as prominent active sites.

The LibDock scores of OXA-1, SHV-1, TEM-1, and CTX-M-15 proteins are presented in Table 3. The best OXA-1, SHV-1, TEM-1, and CTX-M-15 clindamycin and OXA-1, SHV-1, TEM-1, and CTX-M-15-piperacillin tazobactam complexs have been deposited in PMDB and PMDB-ID has been obtained (Table 3).

**Table 3:** Molecular docking calculation of OXA-1, SHV-1, TEM-1, and CTX-M-15 proteins with clindamycin and piperacillin-tazobactam.

Proteins	Drug	PMDB-ID	Absolute Energy	Conf- No.	Relative Energy	LibDock Score
OXA-1	Clindamycin	PM0078676	40.9053	59	10.1825	117.997
	Piperacillin-tazobactam	PM0078677	55.519	2	1.006	153.322
SHV-1	Clindamycin	PM0078678	49.0649	53	18.3421	124.517
	Piperacillin-tazobactam	PM0078679	58.9188	28	4.4058	155.258
TEM-1	Clindamycin	PM0078680	37.8714	15	7.14862	84.4853
	Piperacillin-tazobactam	PM0078681	58.8299	27	4.31684	68.6161
CTX-M-15	Clindamycin	PM0078866	41.0630	17	14.1256	122.556
	Piperacillin-tazobactam	PM0078867	57.1310	8	3.15691	165.589

### Conclusions

The present study shows that multiplex PCR assay may be used for the simultaneous screening of *E. coli* and associated ESBL genes (*bla*_TEM_, *bla*_SHV,_ and *bla*_OXA_) in DFU of a large number of clinical specimens especially in laboratories/hospitals having moderate resources. Findings of this study demonstrate the high prevalence of ESBL-producing *E. coli* in DFUs patients. Multiplex PCR assay showed the highest occurrence of *bla*_CTX-M_ like gene (62.5%) followed by *bla*_TEM_, *bla*_OXA,_ and *bla*_SHV_ genes among the *E. coli* strains isolated from DFUs patients. In view of high prevalence of *bla*_CTX-M_ like gene, it is recommended that multiplex PCR may be routinely used for the screening of this gene in ESBL-producing bacteria. Our findings also showed that majority of the ESBL-producing strains were resistant to β-lactams but showed 100% sensitivity to clindamycin and piperacillin-tazobactam. Furthermore, the 3D models of most prevalent variants of β- lactamases *viz*. TEM-1, SHV-1, OXA-1, and ESBL *viz*. CTX-M-15 were predicted and docking studies with clindamycin and piperacillin/tazobactam were performed. The docking scores of TEM-1, SHV-1, OXA-1, and CTX-M-15 proteins with clindamycin and piperacillin-tazobactam showed significant interaction with active binding residues. The docking studies also revealed that TYR^48^, GLU^69^, SER^70^, GLN^79^, and THR^120^ of OXA-1; THR^40^, ARG^212^, and ILE^214^ of SHV-1; SER^56^, and LEU^172^ residues of TEM-1 protein; PRO^163^ residue of CTX-M-15 form hydrogen bonds with the side chain along with main chain interaction with above drugs. The results of the present study may provide useful insights for developing new antibiotic drugs to minimize ESBLs-mediated resistance problem of bacteria in DFU of diabetic patients.

## Supporting Information

Figure S1 **Phylogenetic analysis of** ***bla*** **_OXA-1_ like gene based on sequences obtained from NCBI database.** **(a)**-Multiple sequence alignment of *bla*_OXA-1_ gene sequence of *E. coli* DF39TA with other species and, **(b)**-phylogenetic tree showing similarity of *E. coli* DF39TA *bla*_OXA-1_ like gene sequence with closely related species.
(DOC)

Figure S2 **Phylogenetic analysis of** ***bla*** **_SHV-1_ like gene based on sequences obtained from NCBI database. (a)**-Alignment of *bla*_SHV-1_ gene sequence of *E. coli* DF39TA with other species, and **(b)**-phylogenetic tree showing similarity of *E. coli* DF39TA *bla*_SHV-1_ like gene sequence with other species.
(DOC)

Figure S3 **Phylogenetic analysis of** ***bla*** **_TEM-1_ like gene based on sequences obtained from NCBI database. (a)**-Alignment of *E. coli* DF39TA *bla*_TEM-1_ gene sequence with other species, and **(b)**-construction of phylogenetic tree showing relatedness of *E. coli* DF39TA *bla*_TEM-1_ like gene sequence with other species.
(DOC)

Figure S4 **Phylogenetic analysis of** ***bla*** **_CTX-M-15_ like gene based on sequences obtained from NCBI database. (a)**-Alignment of *bla*_CTX-M-15_ gene sequence of *E. coli* DF39TA with other species, and **(b)**-phylogenetic tree showing relationship of *E. coli* DF39TA *bla*_CTX-M-15_ like gene sequence with other species.
(DOC)

Figure S5 **Motif elucidation based on multilevel consensus sequence. (a)**- Representative motif elucidation of OXA-1, SHV-1, TEM-1, and CTX-M-15 proteins, and **(b)**-multilevel consensus sequences for the MEME defined motifs observed in OXA-1, SHV-1, TEM-1, and CTX-M-15 proteins from *E. coli* DF39TA.
(DOC)

Figure S6 **Secondary structure of various proteins.** **(a)** - OXA-1, **(b)** - SHV-1, **(c)** - TEM-1, and **(d)** - CTX-M-15 proteins. **(e)** - represents key.
(DOC)

Table S1 **Details of accession number of variants of** ***bla*** **_TEM_,** ***bla*** **_SHV_,** ***bla*** **_CTX-M,_ and** ***bla*** **_OXA_ genes of** ***E. coli*** **strains.**
(DOC)

Table S2 **Modeling and simulation details of OXA** ***-*** **1, SHV-1, TEM-1, and CTX-M-15 proteins.**
(DOC)

Table S3 **Stereo-chemical details of predicted models of OXA** ***-*** **1, SHV-1, TEM-1, and CTX-M-15 proteins using RAMPAGE.**
(DOC)

Table S4 **Model quality estimation of OXA** ***-*** **1, SHV-1, TEM-1, and CTX-M-15 proteins using QMEAN (** http://swissmodel.expasy **. org/qmean/cgi/index.cgi; ).**
(DOC)

Table S5 **Secondary compositional study using VADAR (** http://vadar.wishartlab.com/ **; ).**
(DOC)

Table S6 **Ten predicted active binding sites for OXA** ***-*** **1, SHV-1, TEM-1, and CTX-M-15 using Q-SiteFinder.**
(DOC)
