# Supplementary material for: Detection of Escherichia coli and Associated β-Lactamases Genes from Diabetic Foot Ulcers by Multiplex PCR and Molecular Modeling and Docking of SHV-1, TEM-1, and OXA-1 β-Lactamases with Clindamycin and Piperacillin-Tazobactam
Source: PLoS One. 2013 Jul 4;8(7):e68234. doi: 10.1371/journal.pone.0068234 (PMC3701671; doi:10.1371/journal.pone.0068234)
Supplement: Figure S1 — Phylogenetic analysis of bla OXA-1 like gene based on sequences obtained from NCBI database. (a)-Multiple sequence alignment of bla OXA-1 gene sequence of E. coli DF39TA with other species and, (b)-phylogenetic tree showing similarity of E. coli DF39TA bla OXA-1 like gene sequence with closely related species. (DOC) [file pone.0068234.s001.doc]

**
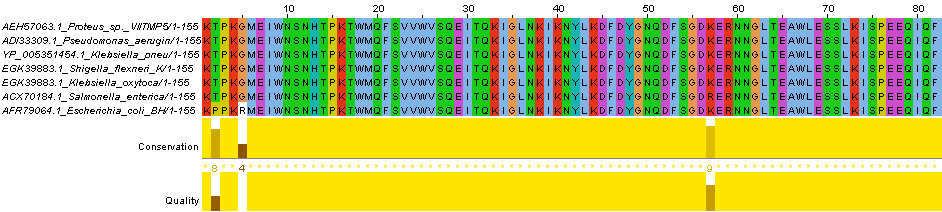
**

**(a)**

**(b)**

**Figure S1.** **Phylogenetic analysis of** *bla*_OXA-1_ **like gene based on sequences obtained from NCBI database.** **(a)**-Multiple sequence alignment of *bla*_OXA-1_ gene sequence of *E. coli* DF39TA with other species, and **(b)**-phylogenetic tree showing similarity of *E. coli* DF39TA *bla*_OXA-1_ like gene sequence with closely related species.
