# Supplementary material for: Detection of Escherichia coli and Associated β-Lactamases Genes from Diabetic Foot Ulcers by Multiplex PCR and Molecular Modeling and Docking of SHV-1, TEM-1, and OXA-1 β-Lactamases with Clindamycin and Piperacillin-Tazobactam
Source: PLoS One. 2013 Jul 4;8(7):e68234. doi: 10.1371/journal.pone.0068234 (PMC3701671; doi:10.1371/journal.pone.0068234)
Supplement: Figure S3 — Phylogenetic analysis of bla TEM-1 like gene based on sequences obtained from NCBI database. (a)-Alignment of E. coli DF39TA bla TEM-1 gene sequence with other species, and (b)-construction of phylogenetic tree showing relatedness of E. coli DF39TA bla TEM-1 like gene sequence with other species. (DOC) [file pone.0068234.s003.doc]

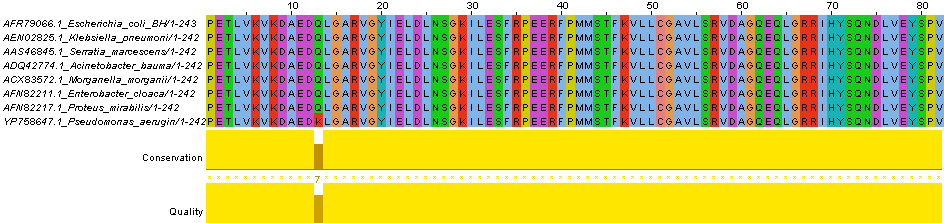


**(a)**

**(b)**

**Figure S3. Phylogenetic analysis of *bla*_TEM-1_ like gene based on sequences obtained from NCBI database. (a)**-Alignment of *E. coli* DF39TA *bla*_TEM-1_ gene sequence with other species, and **(b)**-construction of phylogenetic tree showing relatedness of *E. coli* DF39TA *bla*_TEM-1_ like gene sequence with other species.
