# Supplementary material for: Detection of Escherichia coli and Associated β-Lactamases Genes from Diabetic Foot Ulcers by Multiplex PCR and Molecular Modeling and Docking of SHV-1, TEM-1, and OXA-1 β-Lactamases with Clindamycin and Piperacillin-Tazobactam
Source: PLoS One. 2013 Jul 4;8(7):e68234. doi: 10.1371/journal.pone.0068234 (PMC3701671; doi:10.1371/journal.pone.0068234)
Supplement: Figure S4 — Phylogenetic analysis of bla CTX-M-15 like gene based on sequences obtained from NCBI database. (a)-Alignment of bla CTX-M-15 gene sequence of E. coli DF39TA with other species, and (b)-phylogenetic tree showing relationship of E. coli DF39TA bla CTX-M-15 like gene sequence with other species. (DOC) [file pone.0068234.s004.doc]

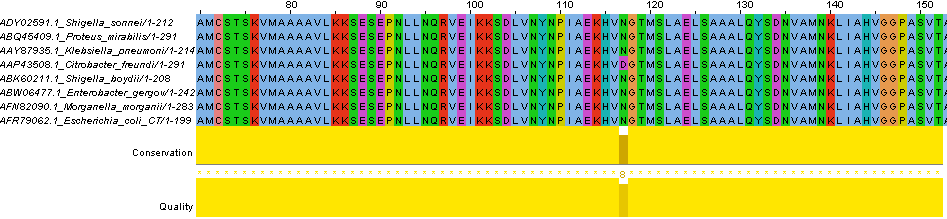


**(a)**


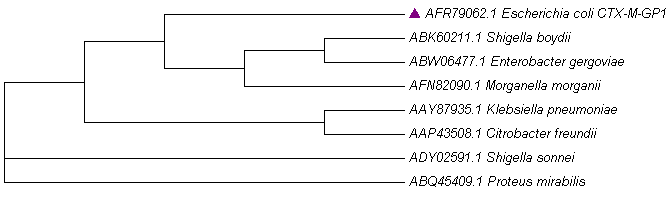


**(b)**

**Figure S4. Phylogenetic analysis of *bla*_CTX-M-15_ like gene based on sequences obtained from NCBI database. (a)**-Alignment of *bla*_CTX-M-15_ gene sequence of *E. coli* DF39TA with other species, and **(b)**-phylogenetic tree showing relationship of *E. coli* DF39TA *bla*_CTX-M-15_ like gene sequence with other species.
