# Supplementary material for: Detection of Escherichia coli and Associated β-Lactamases Genes from Diabetic Foot Ulcers by Multiplex PCR and Molecular Modeling and Docking of SHV-1, TEM-1, and OXA-1 β-Lactamases with Clindamycin and Piperacillin-Tazobactam
Source: PLoS One. 2013 Jul 4;8(7):e68234. doi: 10.1371/journal.pone.0068234 (PMC3701671; doi:10.1371/journal.pone.0068234)
Supplement: Figure S5 — Motif elucidation based on multilevel consensus sequence. (a)- Representative motif elucidation of OXA-1, SHV-1, TEM-1, and CTX-M-15 proteins, and (b)-multilevel consensus sequences for the MEME defined motifs observed in OXA-1, SHV-1, TEM-1, and CTX-M-15 proteins from E. coli DF39TA. (DOC) [file pone.0068234.s005.doc]

**
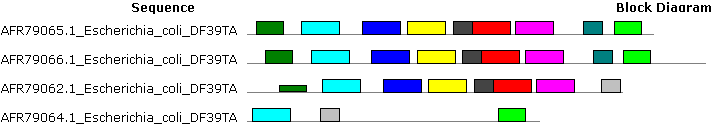
**


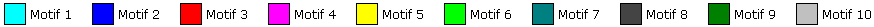


**(a)**

**Motif Width Best possible match**

1 20 RPDERFPMMSTFKVWLCGAV

2 20 RRIHYKQNDLVDYSPVSEKH

3 20 FLRQIGDHVTRLDRWEPELN

4 20 PGDPRDTTSPAAMAQTLRKL

5 20 MTVGELCAAAITMSDNTAAN

6 14 PAGWFIADKSGHGE

7 10 WMEDDKVAGP

8 10 HVGGPAGLTA

9 14 EDQLGGRVGYIEMD

10 10 KNWLKGHDYG

**(b)**

**Figure S5. Motif elucidation based on multilevel consensus sequence. (a)**-Representative motif elucidation of OXA-1, SHV-1, TEM-1, and CTX-M-15 proteins, and **(b)**-multilevel consensus sequences for the MEME defined motifs observed in OXA-1, SHV-1, TEM-1, and CTX-M-15 proteins from *E. coli* DF39TA.
