# Supplementary material for: Detection of Escherichia coli and Associated β-Lactamases Genes from Diabetic Foot Ulcers by Multiplex PCR and Molecular Modeling and Docking of SHV-1, TEM-1, and OXA-1 β-Lactamases with Clindamycin and Piperacillin-Tazobactam
Source: PLoS One. 2013 Jul 4;8(7):e68234. doi: 10.1371/journal.pone.0068234 (PMC3701671; doi:10.1371/journal.pone.0068234)
Supplement: Figure S6 — Secondary structure of various proteins. (a) - OXA-1, (b) - SHV-1, (c) - TEM-1, and (d) - CTX-M-15 proteins. (e) - represents key. (DOC) [file pone.0068234.s006.doc]

| [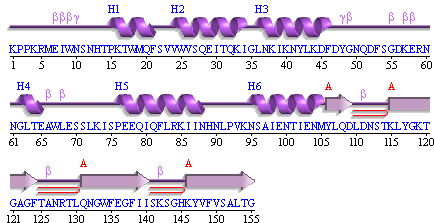](http://www.ebi.ac.uk/thornton-srv/databases/cgi-bin/pdbsum/GetPage.pl?pdbcode=g740&pdb_type=UPLOAD&code=064308&template=wirlarge.html&r=wiring&l=1&large=TRUE&chain=)  **(a)** | [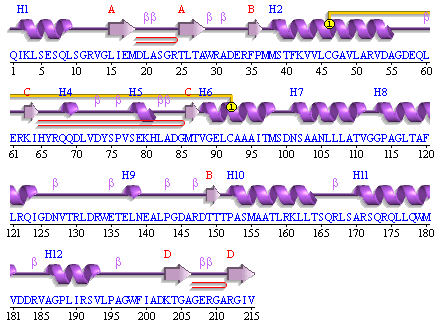](http://www.ebi.ac.uk/thornton-srv/databases/cgi-bin/pdbsum/GetPage.pl?pdbcode=g741&pdb_type=UPLOAD&code=065146&template=wirlarge.html&r=wiring&l=1&large=TRUE&chain=)  **(b)** |
| --- | --- |
| [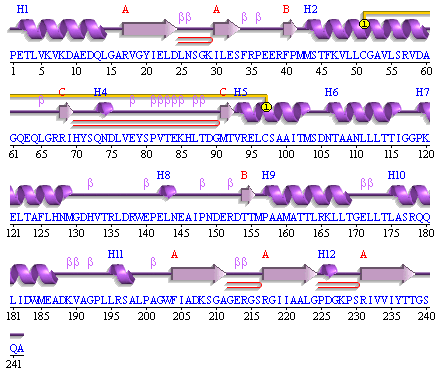](http://www.ebi.ac.uk/thornton-srv/databases/cgi-bin/pdbsum/GetPage.pl?pdbcode=g742&pdb_type=UPLOAD&code=063648&template=wirlarge.html&r=wiring&l=1&large=TRUE&chain=)  **(c)** | 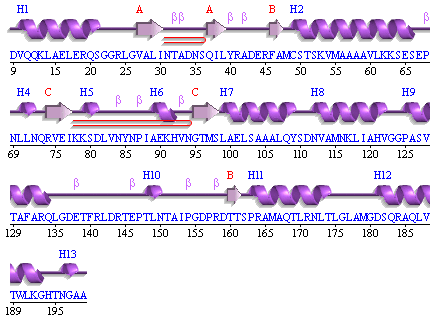  **(d)** |
| 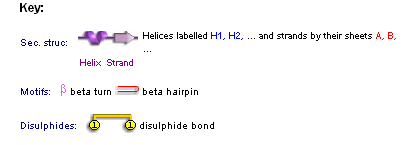  **(e)** | |

**Figure S6. Secondary structure of various proteins.** **(a)** - OXA-1, **(b)** - SHV-1, **(c)** - TEM-1, and **(d)** - CTX-M-15 proteins. **(e)** - represents key.
