# Supplementary material for: Detection of Escherichia coli and Associated β-Lactamases Genes from Diabetic Foot Ulcers by Multiplex PCR and Molecular Modeling and Docking of SHV-1, TEM-1, and OXA-1 β-Lactamases with Clindamycin and Piperacillin-Tazobactam
Source: PLoS One. 2013 Jul 4;8(7):e68234. doi: 10.1371/journal.pone.0068234 (PMC3701671; doi:10.1371/journal.pone.0068234)
Supplement: Table S1 — Details of accession number of variants of bla TEM, bla SHV, bla CTX-M, and bla OXA genes of E. coli strains. (DOC) [file pone.0068234.s007.doc]

**Table S1**. Details of accession number of variants of *bla*_TEM_, *bla*_SHV_, *bla*_CTX-M_, and *bla*_OXA_ genes of *E. coli* strains.

| ***E. coli* strains** | **Variants *bla* genes** | **Accession no.** | ***E. coli* Strains** | **Variants *bla* genes** | **Accession no.** |
| --- | --- | --- | --- | --- | --- |
| DF5SC | TEM-1 | KC859391 | DF7SA | CTX-M -15 | KC859407 |
| DF6SA | TEM-1 | KC859392 | DF9SB | CTX-M -15 | KC859408 |
| DF9SB | TEM -1 | KC859393 | DF29TA | CTX-M -15 | KC859409 |
| DF39TA | TEM -1 | AFR79066 | DF30TA | CTX-M -15 | KC859410 |
| DF49SA | TEM -1 | KC859394 | DF30TD | CTX-M-15 | AFR79061 |
| DF40TA | TEM -10 | KC859395 | DF39TA | CTX-M -15 | AFR79062 |
| DF29TA | TEM-20 | KC859396 | DF49SA | CTX-M-3 | KC859411 |
| DF30TA | TEM-20 | KC859397 | DF13TB | CTX-M-1 | KC859412 |
| DF30TD | TEM-52 | KC859398 | DF5SC | CTX-M-9 | KC859413 |
| DF18SA | SHV -1 | KC859399 | DF5SC | OXA-1 | KC859414 |
| DF30TA | SHV-1 | KC859400 | DF6SA | OXA-1 | KC859415 |
| DF30TD | SHV-1 | KC859401 | DF9SB | OXA-1 | KC859416 |
| DF39TA | SHV-1 | AFR79065 | DF39TA | OXA-1 | AFR79064 |
| DF13TB | SHV-5 | KC859402 | DF18SA | OXA-1 | KC859417 |
| DF5SC | SHV-12 | KC859403 | DF29TA | OXA-1 | KC859418 |
| DF7SA | SHV -12 | KC859404 | DF49SA | OXA-1 | KC859419 |
| DF40TA | SHV-2 | KC859405 | DF7SA | OXA-1 | KC859420 |
| DF6SA | CTX-M-15 | KC859406 | DF10TB | OXA-1 | KC859421 |
