# Supplementary material for: Detection of Escherichia coli and Associated β-Lactamases Genes from Diabetic Foot Ulcers by Multiplex PCR and Molecular Modeling and Docking of SHV-1, TEM-1, and OXA-1 β-Lactamases with Clindamycin and Piperacillin-Tazobactam
Source: PLoS One. 2013 Jul 4;8(7):e68234. doi: 10.1371/journal.pone.0068234 (PMC3701671; doi:10.1371/journal.pone.0068234)
Supplement: Table S2 — Modeling and simulation details of OXA - 1, SHV-1, TEM-1, and CTX-M-15 proteins. (DOC) [file pone.0068234.s008.doc]

**Table S2.** Modeling and simulation details of OXA*-*1, SHV-1, TEM-1, and CTX-M-15 proteins.

| **Proteins** | **Forcefield** | **Potential Energy (kcal/mol)** | **Van der Waals Energy (kcal/mol)** | **Electrostatic Energy (kcal/mol)** | **RMS Gradient (kcal/mol)** |
| --- | --- | --- | --- | --- | --- |
| OXA-1 | CHARMm | -535.146 | 3549.67 | -5659.92 | 309.44 |
| SHV-1 | CHARMm | 344.581 | 6152.20 | -7887.66 | 460.56 |
| TEM-1 | CHARMm | 5148.988 | 11917.21 | -9139.24 | 1089.78 |
| CTX-M-15 | CHARMm | 242.213 | 5814.10 | -7430.49 | 498.385 |
