# Supplementary material for: Detection of Escherichia coli and Associated β-Lactamases Genes from Diabetic Foot Ulcers by Multiplex PCR and Molecular Modeling and Docking of SHV-1, TEM-1, and OXA-1 β-Lactamases with Clindamycin and Piperacillin-Tazobactam
Source: PLoS One. 2013 Jul 4;8(7):e68234. doi: 10.1371/journal.pone.0068234 (PMC3701671; doi:10.1371/journal.pone.0068234)
Supplement: Table S3 — Stereo-chemical details of predicted models of OXA - 1, SHV-1, TEM-1, and CTX-M-15 proteins using RAMPAGE. (DOC) [file pone.0068234.s009.doc]

**Table S3.** Stereo-chemical details of predicted models of OXA*-*1, SHV-1, TEM-1, and CTX-M-15 proteins using RAMPAGE.

| **Proteins** | **% of residues in favoured region** | **% of residues in allowed region** | **% of residues in outlier region** |
| --- | --- | --- | --- |
| OXA-1 | 93.5 | 6.5 | 0.0 |
| SHV-1 | 98.1 | 1.9 | 0.0 |
| TEM-1 | 98.3 | 1.2 | 0.4 |
| CTX-M-15 | 98.9 | 1.1 | 0.0 |
