# Supplementary material for: Detection of Escherichia coli and Associated β-Lactamases Genes from Diabetic Foot Ulcers by Multiplex PCR and Molecular Modeling and Docking of SHV-1, TEM-1, and OXA-1 β-Lactamases with Clindamycin and Piperacillin-Tazobactam
Source: PLoS One. 2013 Jul 4;8(7):e68234. doi: 10.1371/journal.pone.0068234 (PMC3701671; doi:10.1371/journal.pone.0068234)
Supplement: Table S4 — Model quality estimation of OXA - 1, SHV-1, TEM-1, and CTX-M-15 proteins using QMEAN ( http://swissmodel.expasy . org/qmean/cgi/index.cgi; ). (DOC) [file pone.0068234.s010.doc]

**Table S4**. Model quality estimation of OXA*-*1, SHV-1, TEM-1, and CTX-M-15 proteins using QMEAN (http://swissmodel.expasy. org/qmean/cgi/index.cgi;).

| **Proteins** | **QMEAN score** | **Global Z-score** |
| --- | --- | --- |
| OXA-1 | 0.63 | -1.28 |
| SHV-1 | 0.817 | 0.49 |
| TEM-1 | 0.859 | 0.95 |
| CTX-M-15 | 0.76 | -0.07 |
