# Supplementary material for: Detection of Escherichia coli and Associated β-Lactamases Genes from Diabetic Foot Ulcers by Multiplex PCR and Molecular Modeling and Docking of SHV-1, TEM-1, and OXA-1 β-Lactamases with Clindamycin and Piperacillin-Tazobactam
Source: PLoS One. 2013 Jul 4;8(7):e68234. doi: 10.1371/journal.pone.0068234 (PMC3701671; doi:10.1371/journal.pone.0068234)
Supplement: Table S5 — Secondary compositional study using VADAR ( http://vadar.wishartlab.com/ ; ). (DOC) [file pone.0068234.s011.doc]

**Table S5.** Secondary compositional study using VADAR (http://vadar.wishartlab.com/;).

| **Proteins** | **Helix (%)** | **Beta (%)** | **Coil (%)** | **Turn (%)** |
| --- | --- | --- | --- | --- |
| OXA-1 | 37 | 25 | 37 | 23 |
| SHV-1 | 43 | 16 | 40 | 16 |
| TEM-1 | 40 | 24 | 34 | 23 |
| CTX-M-15 | 48 | 16 | 35 | 29 |
