# Supplementary material for: Detection of Escherichia coli and Associated β-Lactamases Genes from Diabetic Foot Ulcers by Multiplex PCR and Molecular Modeling and Docking of SHV-1, TEM-1, and OXA-1 β-Lactamases with Clindamycin and Piperacillin-Tazobactam
Source: PLoS One. 2013 Jul 4;8(7):e68234. doi: 10.1371/journal.pone.0068234 (PMC3701671; doi:10.1371/journal.pone.0068234)
Supplement: Table S6 — Ten predicted active binding sites for OXA - 1, SHV-1, TEM-1, and CTX-M-15 using Q-SiteFinder. (DOC) [file pone.0068234.s012.doc]

**Table S6**. Ten predicted active binding sites for OXA*-*1, SHV-1, TEM-1, and CTX-M-15 using Q-SiteFinder.

| **Predicted site** | **Site Volume**  **(Cubic Angstrom)** | **Active Site Residues** |
| --- | --- | --- |
| **OXA-1 (Protein Volume: 14589 Cubic Angstroms)** | | |
| 1 | 234 | LYS^16^, THR^17^ ,MET^19^, GLN ^20^, GLU ^103^, ASN ^104^, MET^105^, TYR^106^, LEU^107^, GLY^118^, LYS^119^, THR^120^, PHE ^135^ |
| 2 | 202 | ALA^66^, TRP^67^, LEU^68^, GLU^69^, SER^70^, LEU^72^, LYS^73^, GLY^121^, ALA^122^, GLY^123^, ASN^132^, GLY^133^, TRP^134^ |
| 3 | 196 | THR^31^, ILE^34^, LYS^38^, ILE^39^, TYR^42^, LEU^43^, PHE^46^, TYR^48^, PHE^53^, TRP^67^, LEU^72^, PHE^82^ |
| 4 | 140 | TRP^9^, TRP^18^, MET^19^, SER^22^, VAL^23^, VAL^24^, SER^27^, TRP^67^, LEU^68^, LYS^119^, GLY^121^ |
| 5 | 154 | MET^19^, TYR^48^ , ILE^74^, GLN^79^, PHE^82^, LEU^83^, ILE^86^ , THR^101^, MET^105^, GLY^118^, LYS^119^, GLU^136^, GLY^137^, PHE^138^ |
| 6 | 120 | TYR^42^, PHE^46^, PHE^82^, ILE^86^, LEU^91^, VAL^93^, ALA^97^, ILE ^98^, THR^101^, ILE^102^ |
| 7 | 105 | LEU^36^ LYS^40^, PHE^53^, SER^54^, GLY^55^, ASP^56^, LYS^57^, ASN^60^, ASN^61^, GLY^62^, LEU^63^ |
| 8 | 97 | TYR^48^, GLN^79^, LEU^83^, LYS^119^, THR^120^ , GLY^121^, TRP^134^, PHE^135^, GLU^136^ |
| 9 | 104 | ARG^84^, ILE^87^, ASN^88^, PHE^138^, LYS^146^, TYR^147^, VAL^148^ |
| 10 | 86 | SER^75^, PRO^76^, ASN^132^, GLY^133^, TRP^134^, ALA^152^, LEU^153^, THR^154^ |
| **SHV-1 (Protein Volume: 19837 Cubic Angstroms)** | | |
| 1 | 486 | ARG^12^, VAL^13^, GLY^14^, LEU^15^, ILE^16^, PHE^35^, PRO^36^, MET^37^, MET^38^, THR^40^,THR^150^,THR^151^, PRO^152^, MET^155^, ALA^156^, LEU^159^, ARG^209^,GLY^210^, ALA^211^, ARG^212^, GLY^213^, ILE^214^, VAL^215^ |
| 2 | 203 | ILE^16^, GLU^17^, MET^18^, ASP^19^, LEU^20^, ALA^156^, ALA^157^, LEU^159^, ARG^160^, LEU^163^, LEU^177^, MET^180^, ALA^201^ |
| 3 | 155 | ARG^34^, PHE^35^, PRO^36^, ALA^141^, LEU^142^, PRO^143^, GLY^144^, ASP^145^ ALA^146^ , THR^149^, GLU^208^, ARG^209^ |
| 4 | 122 | ALA^48^, ALA^51^, ARG^52^, VAL^111^, GLY^116^, LEU^117^, ALA^119^, PHE^120^, GLN^123^ |
| 5 | 106 | LEU^20^, LEU^163^, THR^164^, ALA^170^, GLN^173^, ARG^174^, LEU^177^ |
| 6 | 97 | GLY^125^, ASP^126^, ASN^127^, VAL^128^, ALA^153^, SER^154^, MET^155^, ALA^157^, THR^158^, LYS^161^ |
| 7 | 97 | THR^136^, GLU^137^, LEU^138^, ASN^139^, GLU^140^, GLU^208^ |
| 8 | 117 | ALA^51^, ASP^54^, PHE^120^, GLN^123^, ILE^124^, LEU^162^, ARG^167^, LEU^168^ |
| 9 | 98 | PRO^114^, ALA^115^, THR^118^, ARG^122^, ARG^130^, LEU^131^, ASP^132^ |
| 10 | 84 | VAL^77^, MET^86^, GLU^90^, ALA^93^, ALA^94^, THR^97^, MET^98^, ARG^184^ |
| **TEM -1 (Protein Volume: 22555 Cubic Angstroms)** | | |
| 1 | 239 | ARG^17^, ARG^39^, PHE^40^, PRO^41^, ARG^138^, LEU^43^, ASN^144^, GLU^145^, ALA^146^, ILE^147^, PRO^148^, ASN^149^, ASP^150^, GLU^151^, ARG^152^, ASP^153^, THR^154^, THR^238^ |
| 2 | 209 | SER^44^, LYS^47^, TYR^79^, SER^104^, ASN^106^, GLU^140^, ASN^144^, VAL^190^, ALA^191^, LYS^208^, SER^209^, GLY^210^, ALA^211^, ARG^217^ |
| 3 | 216 | ALA^53^, VAL^54^, SER^56^, ARG^57^, GLN^62^, THR^115^, ILE^116^, GLU^121^, LEU^122^, ALA^124^, PHE^125^, ASN^128^ |
| 4 | 118 | GLU^37^, GLY^130^, ASP^131^, HIS^132^, VAL^133^, MET^156^, ALA^158^, ALA^159^, THR^162^, THR^163^ |
| 5 | 119 | GLU^78^, ARG^138^, PRO^141^, GLU^142^, LEU^143^, ASN^144^, GLU^145^, GLU^213^ |
| 6 | 92 | LEU^55^, VAL^58^, ASP^59^, LEU^65^, ARG^94^, THR^174^, ALA^176^, SER^177^, GLN^180^ |
| 7 | 90 | ASN^128^, MET^129^, LYS^166^, LEU^167^, GLY^170^, GLU^171^, LEU^172^ |
| 8 | 94 | PRO^119^, LYS^120^, THR^123^, ARG^135^, LEU^136^, ASP^137^ |
| 9 | 84 | LEU^194^, LEU^195^, GLY^210^, ARG^217^, GLY^218^, ILE^219^, ILE^235^, THR^237^ |
| 10 | 84 | THR^3^, LYS^6^, VAL^7^, ALA^10^, VAL^18^, TYR^20^, ILE^235^ |
| **CTX-M-15 (Protein Volume: Cubic Angstroms)** | | |
| 1 | 255 | ARG^24^, LEU^25^, GLY^26^, VAL^27^, ALA^28^, GLU^44^, PHE^46^, ALA^47^, MET^48^, THR^51^, THR^161^, SER^162^, PRO^163^, ARG^164^, MET^166^, ALA^167^, LEU^170^ |
| 2 | 145 | ARG^24^, ARG^45^, PHE^46^ , ALA^47^, CYS^49^, ALA^152^, ILE^153^, PRO^154^, GLY^155^, ASP^156^, ARG^158^, THR^160^ |
| 3 | 109 | MET^48^, THR^51^, SER^52^, LYS^53^, VAL^54^, MET^55^, PHE^140^, ALA^165^, MET^166^, THR^169^, LEU^170^, LEU^173^ |
| 4 | 107 | SER^50^, THR^51^, LYS^53^, VAL^54^, ALA^106^, LEU^107^, SER^110^, HIS^194^, ASN^196^ |
| 5 | 122 | ALA^28^, LEU^29^, ILE^30^, THR^32^, ALA^167^, LEU^170^, ARG^171^, THR^174^, LEU^175^ |
| 6 | 121 | ALA^59^, VAL^60^, LYS^62^, LYS^63^, VAL^122^, SER^127^, ALA^130^, PHE^131^,GLN^134^, |
| 7 | 142 | GLN^73^, VAL^75^, GLU^76^, LYS^78^, ASP^81^, LEU^99^, VAL^113^, ASN^116^, LYS^117^, ALA^120^, HIS^121^ |
| 8 | 105 | ILE^88^, HIS^92^, MET^97^, GLU^101^, LEU^102^, ALA^104^, ALA^105^, GLN^108^, TYR^109^ |
| 9 | 97 | LEU^82^,VAL^83^, ASN^84^, TYR^85^, ASN^86^, PRO^87^, GLU^90^ |
| 10 | 92 | ARG^24^, GLU^44^, ARG^45^, PHE^46^, ALA^47^, ILE^153^, PRO^154^, GLY^155^, ASP^156^, PRO^157^ |
